# Alpha6-Integrin Regulates FGFR1 Expression through the ZEB1/YAP1 Transcription Complex in Glioblastoma Stem Cells Resulting in Enhanced Proliferation and Stemness

**DOI:** 10.3390/cancers11030406

**Published:** 2019-03-22

**Authors:** Aline KOWALSKI-CHAUVEL, Valerie GOUAZE-ANDERSSON, Laurent BARICAULT, Elodie MARTIN, Caroline DELMAS, Christine TOULAS, Elizabeth COHEN-JONATHAN-MOYAL, Catherine SEVA

**Affiliations:** 1 INSERM UMR.1037-Cancer Research Center of Toulouse (CRCT)/University Paul Sabatier Toulouse III, 31037 Toulouse, France; aline.chauvel@inserm.fr (A.K.-C.); valerie.gouaze-andersson@inserm.fr (V.G.-A.); laurent.baricault@inserm.fr (L.B.); delmas.caroline@iuct-oncopole.fr (C.D.); toulas.christine@iuct-oncopole.fr (C.T.); moyal.elisabeth@iuct-oncopole.fr (E.C.-J.-M.); 2 IUCT-Oncopole Toulouse, 31037 Toulouse, France; martin.elodie@iuct-oncopole.fr

**Keywords:** glioblastoma, signaling, FGFR1, α 6-integrin, cancer stem cells

## Abstract

Glioblastoma (GBM) is the most lethal primary brain tumor in adults and is known to be particularly aggressive and resistant to anti-cancer therapies, mainly due to the presence of GBM stem cells (GBMSC). By in vitro approaches supported by analysis from patients’ databases, we determined how α6-integrin and Fibroblast Growth Factor Receptor 1 (FGFR1) work in concert to regulate proliferation and stemness of GBMSC. We showed that α6-integrin regulates the expression of FGFR1 and its target gene Fokhead Box M1 (FOXM1) via the ZEB1/YAP1 transcription complex. These results were in accordance with the positive correlation observed in GBM between α6-integrin expression and its target genes ZEB1/YAP1, FGFR1, and FOXM1 in the databases, TCGA and Rembrandt. In addition, the clinical data demonstrate that GBM patients with high levels of the five genes signature, including α6-integrin, ZEB1/YAP1, FGFR1 and FOXM1, have a significantly shorter overall survival. In vitro, we observed a similar decrease in the expression of stemness-related factors, neurospheres forming capacity, as well as spheroids growth when α6-integrin or FGFR1 was blocked individually with specific siRNA, whereas the combination of both siRNA led to a significantly higher inhibition of spheres formation. These data suggest that co-administration of anti-FGFR1 and anti-α6-integrin could provide an improved therapeutic response in GBMSC.

## 1. Introduction

Glioblastoma (GBM) is the most lethal primary brain tumor in adults. Despite treatments including surgical resection, radiotherapy, and adjuvant chemotherapy, median survival remains less than two years and recurrence is practically inevitable [1]. Therefore, treatment strategies that target resistant GBM cells are critically needed. It is speculated that this high recurrence is due to the presence of glioblastoma stem cells (GBMSC), also called GBM initiating cells, which are particularly chemo- and radio-resistant. These GBMSC are characterized by their ability of self-renewal, the overexpression of neural stem cell markers, their pluripotent aptitude to differentiate into neural lineages, and their high tumorigenic potential in vivo [2,3]. Integrins are major receptors involved in cell–matrix adhesion that regulate numerous cellular effects, including proliferation, survival, and invasion [4,5]. They play an important role in resistance to anti-cancer therapies. Among the integrin subunits strongly expressed in GBM, α6 is of particular interest [6,7]. Although α6-integrin is weakly expressed in normal brains, its expression is high in embryonic and adult normal neural stem cells and is involved in the growth regulation of these particular cells [8,9]. In addition, α6-integrin is recognized as an enrichment marker for GBMSC and plays a crucial role in their capacity of self-renewal, proliferation and tumor formation [10].

Fibroblast growth factor receptors (FGFRs), encoded by four genes (FGFR1-4), are involved in a variety of cellular process including stemness, proliferation, or resistance to therapies [11,12]. Increasing evidence demonstrates the importance of FGFRs/FGFs axis in GBM cells and particularly in GBMSC. GBM express high levels of FGFR1 as well as FGFs compared to normal brains [13,14]. In addition, several studies have shown that FGFRs are involved in GBM growth and progression [14,15,16,17]. Specifically, for GBMSC, FGF2 helps to maintain their stem cell state, and inhibition of FGFR1 expression or blocking of FGF2 with antibodies decreases the proliferation of GBMSC [18,19,20,21].

α6-integrin and FGFR1 are both expressed by GBMSC but their potential action in controlling GBMSC behavior have not been linked. Here we hypothesized that α6-integrin and FGFR1 work in concert in GBMSC. In this study, the data show a cross-talk between α6-integrin and FGFR1 signaling pathways. We demonstrate that α6-integrin controls the expression of FGFR1 through the ZEB1 and YAP transcription factors, leading to an increase in the expression of stemness-related factors, neurospheres forming capacity, as well as spheroids growth in GBMSC.

## 2. Results

### 2.1. α6-Integrin Regulates FGFR1 Signaling

To explore the hypothesis of a cross-talk between α6-integrin and FGFRs signaling pathways we used GBMSC derived from GBM biopsy specimens (GC1, GC2) cultured as primary neurospheres that we previously characterized and were known to express high levels of the α6-integrin subunit [22,23].

**Figure 1:**
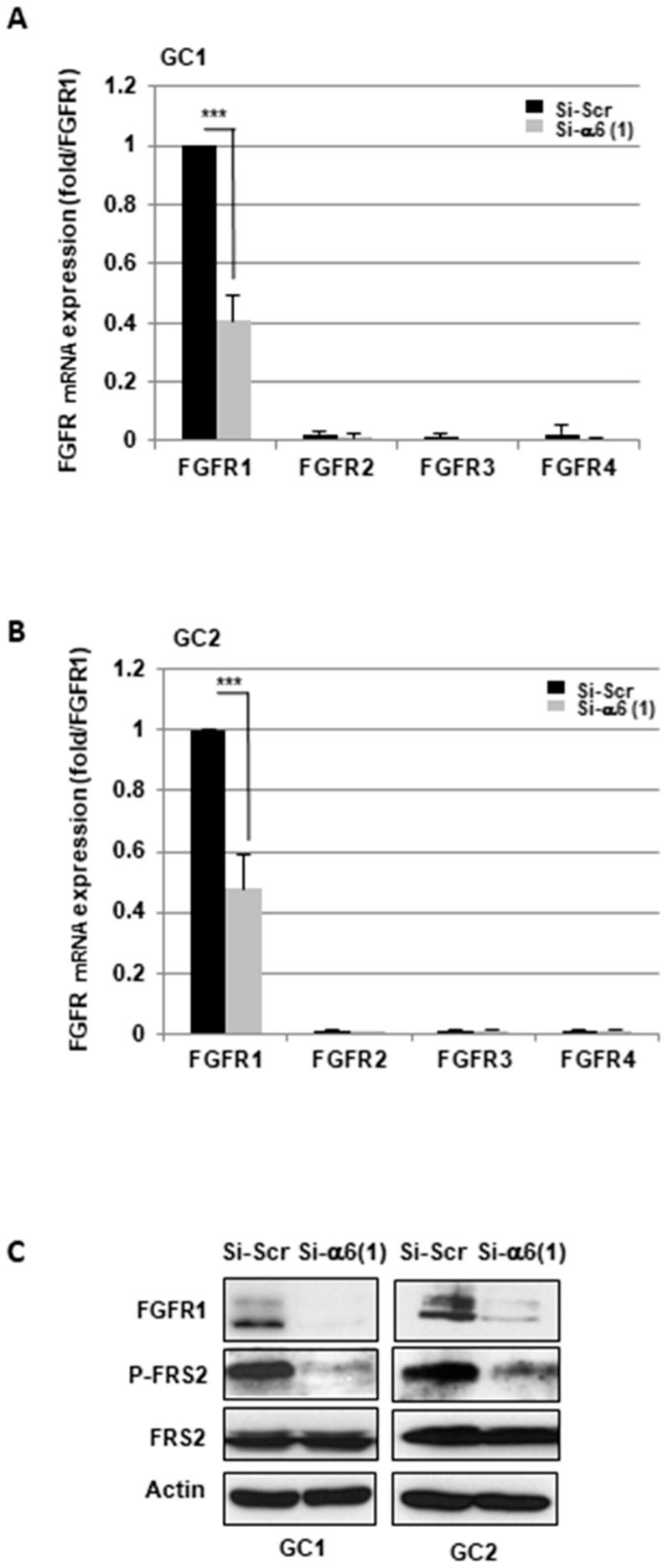
Targeting α6-integrin decreases Fibroblast Growth Factor Receptor 1 (FGFR1) expression and the phosphorylation of its downstream target FGFR substrate 2 (FRS2). (**A**–**C**) Glioblastoma stem cells (GBMSC) derived from Glioblastoma (GBM) biopsy specimens (GC1, GC2) were transfected with an α6-integrin siRNA (si-α6 (1)) or a scramble control (si-Scr). (**A**,**B**) FGFRs expression was analyzed by real-time PCR. Quantifications of 3 independent experiments are presented as means ± SD. *** *p* < 0.001. (**C**) FGFR1 and FRS2 protein expression, as well as the phosphorylation of FRS2 (P-FRS2), were analyzed with Western blot. Images are representative of 3 independent experiments. Actin was used as a loading control.

We first analyzed the role of α6-integrin in FGFRs expression. For this purpose, we used two different specific siRNA (si-α6(1) and si-α6(2)), previously validated to knockdown α6-integrin expression in the GBMSC GC1 and GC2 [22]. As shown in Figure 1A,B, FGFR2, 3, and 4 were very little expressed as compared to FGFR1 in all primary neurospheres, and we decided to focus our study on FGFR1. Both α6-integrin siRNA inhibited significantly FGFR1 mRNA expression compared to a scramble control in the neurospheres GC1 and GC2 (Figure 1A,B and Figure S1A,B). A high inhibition of FGFR1 expression by α6-integrin siRNA was also confirmed at the protein level by Western blot analysis (Figure 1C).

FGFR substrate 2 (FRS2) is a key adaptor protein that is largely specific for FGFRs. The activated FGFRs phosphorylate FRS2 allows the recruitment and activation of downstream signaling pathways [11]. Using a phospho-specific antibody against phosphorylated FRS2, we observed, in correlation with the decrease of FGFR1 expression, a high inhibition of FRS2 phosphorylation when α6-integrin was down-regulated. In contrast, total FRS2 expression was not affected (Figure 1C).

**Figure 2:**
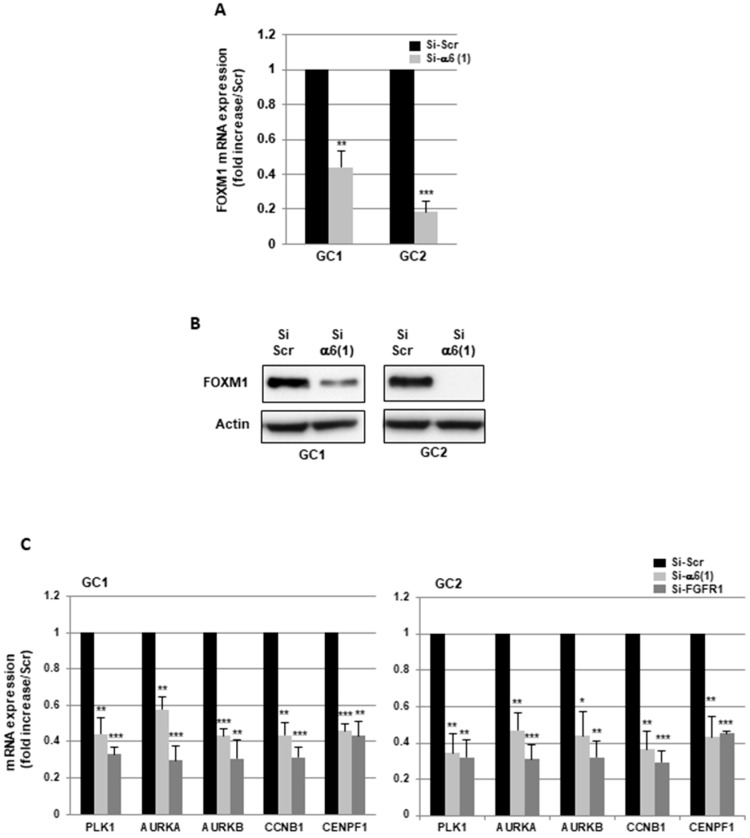
Down-regulation of α6-integrin inhibits the expression of the transcription factor, FOXM1, and its downstream targets. (**A**–**C**) GBMSC derived from GBM biopsy specimens (GC1, GC2) were transfected with an α6-integrin siRNA (si-α6 (1)), a specific FGFR1 siRNA (si-FGFR1), or a scramble control (si-Scr). (**A**,**C**) The expression of FOXM1 and its downstream target genes were analyzed using real-time PCR. Quantifications of 3 independent experiments are presented as means ± SD. *** *p* < 0.001; ** 0.001 < *p* < 0.01; * 0.01 < *p* < 0.05. (**B**) FOXM1 protein expression was analyzed using Western blot. Images are representative of 3 independent experiments. Actin was used as a loading control.

We have previously identified FOXM1 as a target gene of the FGFR1 signaling pathway, using a transcriptomic approach in GC1 and GC2 cells silenced or not for FGFR1 [23]. FOXM1 is a critical proliferation-associated transcription factor which regulates numerous genes involved in processes such as cell cycle control, proliferation, stemness, and tumorigenesis [24,25]. We therefore examined the status of FOXM1 in GBMSC neurospheres when α6-integrin was down-regulated. In cells expressing an α6-integrin siRNA, the expression of FOXM1 was highly decreased at the mRNA and protein levels (Figure 2A,B). In accordance with these results, we also observed, in neurospheres transfected with an α6-integrin siRNA, a significant inhibition of several direct target genes of FOXM1 involved in cell cycle and mitosis, including PLK1, AURKA, AURKB, CCNB1, and CENPF1 (Figure 2C). The same FOXM1 target genes were also down-regulated using a FGFR1 siRNA previously validated in the GBMSC GC1 and GC2 (Figure 2C).

**Figure 3:**
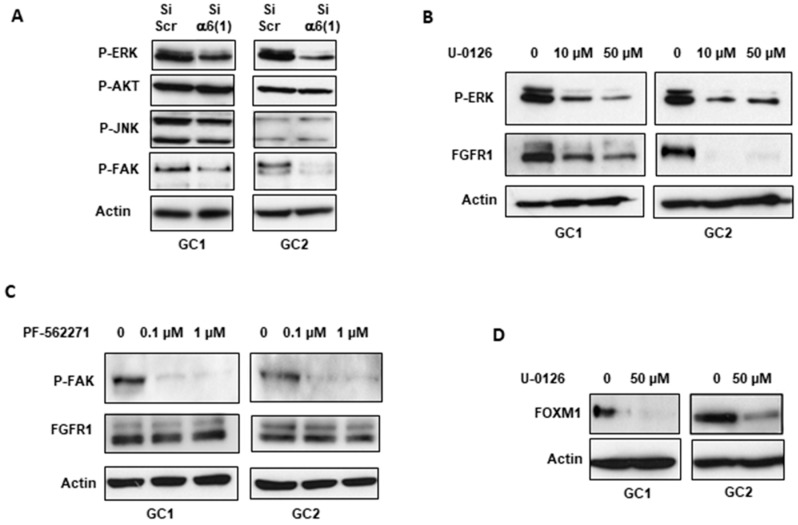
FGFR1 and its downstream target, FOXM1, are regulated via the ERK pathway. (**A**) GBMSC derived from GBM biopsy specimens (GC1, GC2) were transfected with an α6-integrin siRNA (si-α6 (1)) or a scramble control (si-Scr). Activation of the different signaling pathways, ERKs, AKT, JNK or FAK were analyzed with Western blot using phospho-specific antibodies. (**B**–**D**) GBMSC were pretreated or not with 10–50 µM of the ERK inhibitor U-0126 or with 0.1–1 µM of the FAK inhibitor PF-562271 for 24 h. The phosphorylation of ERKs or FAK, as well as the expression of FGFR1, were analyzed using Western blot. Images are representative of 3 independent experiments. Actin was used as a loading control.

Next, we investigated the cellular mechanisms by which α6-integrin regulates FGFR1 expression in GBMSC. Several signaling pathways have been described downstream from integrins activation. They include the ERK, AKT, or JNK pathways, as well as the integrin-associated signaling protein FAK leading to the expression of genes involved in cell cycle regulation, proliferation, and survival [25]. As shown in Figure 3A, only the phosphorylation of FAK and ERKs was inhibited in cells transfected with the α6-integrin siRNA. We therefore analyzed the involvement of these two pathways in FGFR1 expression using two specific inhibitors, U-0126, and PF-562271, that respectively block the ERKs and FAK activation. In cells pre-treated with 10 or 50 µM of the ERKs inhibitor (U-0126), two doses which drastically inhibit the ERKs phosphorylation, we observed a significant decrease in FGFR1 expression in GC1 and GC2 cells (Figure 3B), suggesting a role of the ERKs pathway upstream of FGFR1 expression. As expected, FOXM1 expression was also blocked by the ERKs inhibitor, U-0126 (Figure 3D). In contrast, although 0.1–1 µM of the FAK inhibitor totally blocks FAK phosphorylation, FGFR1 expression was not impacted (Figure 3C). 

### 2.2. α6-Integrin and FGFR1 Cooperate in GBMSC to Regulate Cell Cycle and Proliferation

**Figure 4:**
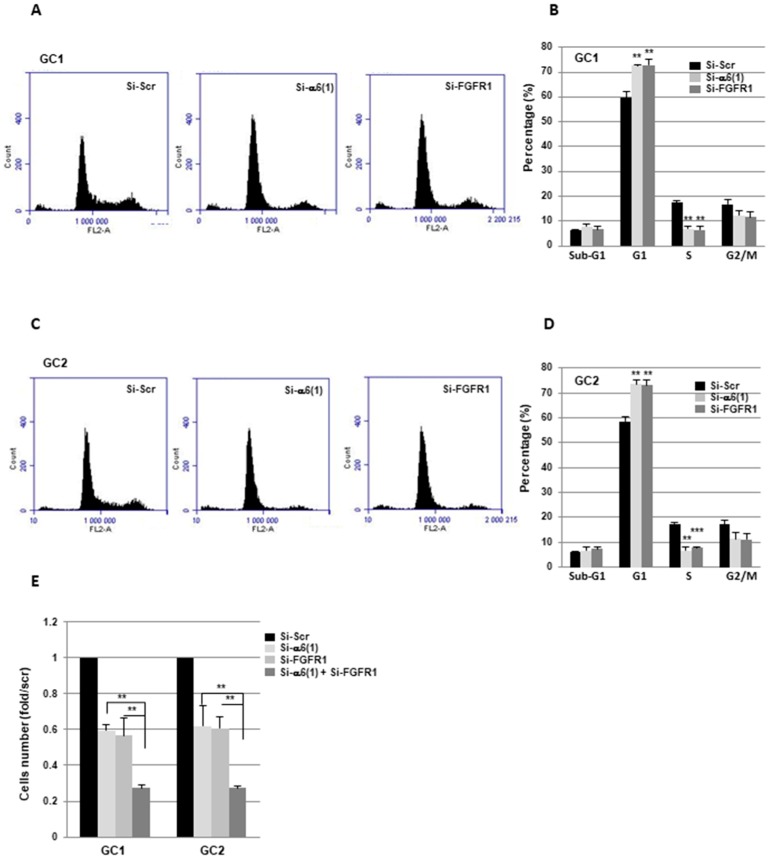
Cell cycle analysis and proliferation. (**A**–**E**) GBMSC derived from GBM biopsy specimens (GC1, GC2) were transfected with an α6-integrin siRNA (si-α6 (1)), a FGFR1 siRNA (si-FGFR1), a combination of both siRNA (si-α6 (1) + si-FGFR1), or a scramble control (si-Scr). Propidium iodide staining was performed as described in section “Materials and Methods”, and the DNA content was analyzed using flow cytometry. The % of cells in sub-G1, G1, S and G2/M phases were quantified by the BD Accuri C6 software. (**E**) Cells number was measured by using the cell counter Countess II FL. Quantifications of 3 independent experiments are presented as means ± SD. *** *p* < 0.001; ** 0.001 < *p* < 0.01.

Since we observed a decrease in the expression of several genes involved in cell proliferation following α6-integrin or FGFR1 inhibition, we then analyzed the cell cycle by flow cytometry. 24h after the transfection of the GBM neurospheres with a siRNA targeting α6-integrin or FGFR1, we observed in GC1 and GC2 an increase in the percentage of cells in the G1 phase and a concomitant decrease in the S phase suggesting a blocking of the cell cycle in G1 (Figure 4A–D). These data are consistent with the significant inhibition of cell proliferation obtained when the expression of α6-integrin or FGFR1 was blocked in GBMSC (Figure 4E). Interestingly, this inhibition was much higher with a dual transfection which blocked efficiently both α6-integrin and FGFR1 expression (Figure 4E and Figure S1C,D). 

### 2.3. α6-Integrin Regulates FGFR1 Expression Via the Transcription Factors ZEB1 and YAP1

**Figure 5:**
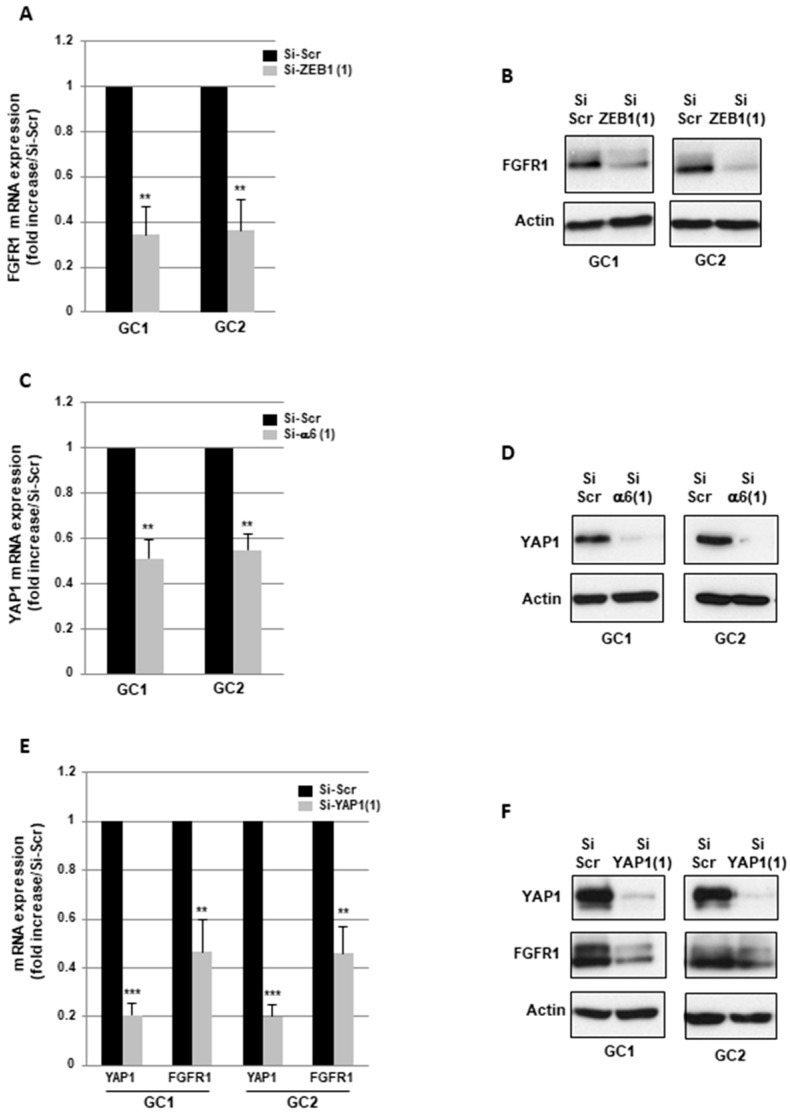
The transcription complex ZEB1/YAP1 regulates FGFR1 expression. (**A**–**F**) GBMSC derived from GBM biopsy specimens (GC1, GC2) were transfected with a ZEB1 siRNA (si-ZEB1(1)), an α6-integrin siRNA (si-α6 (1)), a YAP1 siRNA (si-YAP1) or a scramble control (si-Scr). (**A**,**C**,**E**) the expression of FGFR1 or YAP1 was analyzed by real-time PCR. Quantifications of 3 independent experiments are presented as means ± SD. *** *p* < 0.001; ** 0.001 < *p* < 0.01. (**B**,**D**,**F**) FGFR1 and YAP1 proteins expression were analyzed using Western blot. Images are representative of 3 independent experiments. Actin was used as a loading control.

We have previously published the regulation of the transcription factor Zinc Finger E-Box Binding Homeobox 1 (ZEB1) by α6-integrin in GBMSC [22]. Therefore, to go further into the mechanism by which α6-integrin regulates FGFR1 expression, we down-regulated ZEB1 using two different specific siRNA previously validated in GBMSC GC1 and GC2 [22]. As shown in Figure 5A and Figure S1E, FGFR1 mRNA expression was significantly decreased in cells transfected with both ZEB1 siRNA compared to a scramble control. A high inhibition of FGFR1 expression by a ZEB1 siRNA was also confirmed at the protein level by Western blot analysis (Figure 5B). In addition, the expression of the downstream target of FGFR1, FOXM1, was also inhibited at the mRNA and protein level when ZEB1 was blocked in GBMSC (Figure S2A,B).

Recently, it has been reported that ZEB1 directly binds to the Hippo pathway effector YAP1 and turns into a transcriptional co-activator of common ZEB1/YAP1 target genes involved in proliferation, stemness, or therapy resistance [26]. In Figure 5C,D, we show that blocking α6-integrin expression results in the down-regulation of YAP1 expression at the mRNA and protein level in GBMSC. In addition, we validated specific siRNA which block YAP1 expression (Figure 5E,F) and we observed a significant decrease in FGFR1 expression in GBM neurospheres transfected with the YAP1 siRNA compared to a scrambled control (Figure 5E,F). These results indicate that α6 integrin might control FGFR1 expression by regulating the two partners of the ZEB1/YAP1 complex.

### 2.4. α6-Integrin and FGFR1 Cooperate in GBMSC to Regulate Neurospheres Formation and Stemness

**Figure 6:**
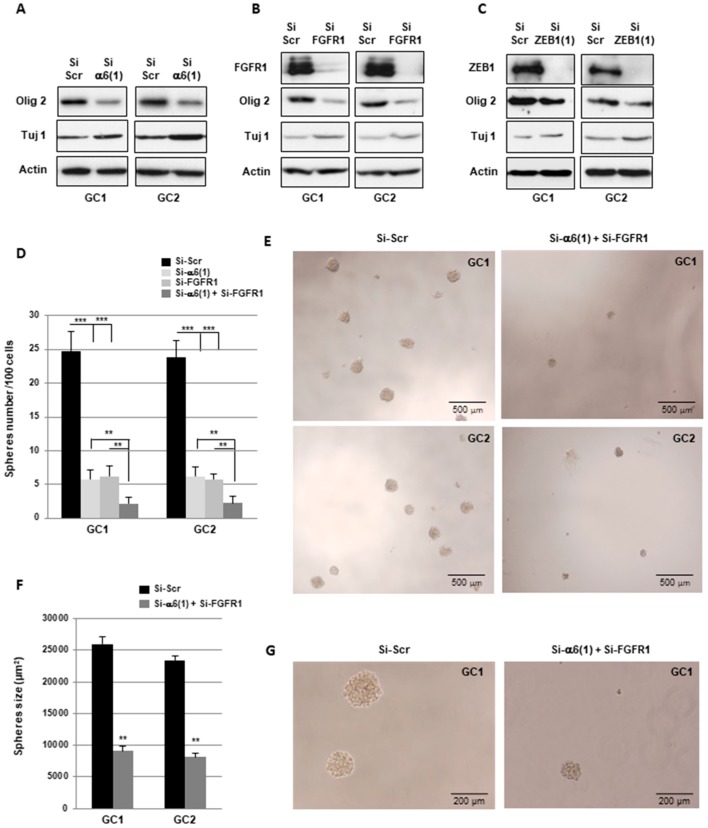
α6-integrin and FGFR1 cooperate in GBMSC to regulate neurospheres formation and stemness. (**A**–**G**) GBMSC derived from GBM biopsy specimens (GC1, GC2) were transfected with an α6-integrin siRNA (si-α6 (1)), a FGFR1 siRNA (si-FGFR1), a combination of both siRNA (si-α6 (1) + si-FGFR1), a scramble control (si-Scr) or a ZEB1 siRNA (si-ZEB1). (**A**–**C**) Olig2, Tuj1, FGFR1, and ZEB1 proteins expression was analyzed using Western blot. Images are representative of 3 independent experiments. Actin was used as a loading control. (**D**–**F**) Spheres formation was analyzed as described in section “Materials and Methods”. Micrographs from representative fields were taken ×20 (**E**), ×40 (**G**). (**C**) neurospheres number was counted under the microscope. (**F**) The spheres size was measured using the Image J software. Quantifications of 3 independent experiments are presented as means ± SD. *** *p* < 0.001; ** 0.001 < *p* < 0.01.

α6-integrin and FGFR1 have been previously shown to contribute to maintaining the stem state of glioblastoma cells [10,19,23]. Here, we confirmed a decrease of the stem cell marker, Olig2, when α6-integrin or FGFR1 were down-regulated in GBMSC, GC1, and GC2, whereas the differentiation marker Tuj-1 increased weakly (Figure 6A,B). In addition, as previously described by Siebzehnrubl et al. [27], Olig2 was also significantly decreased when ZEB1 was inhibited (Figure 6C and Figure S3A).

Neurospheres formation was examined in GBMSC transfected with an α6-integrin siRNA, a FGFR1 siRNA, or the combination of both. Under these conditions, we observed a similar decrease in spheres number when α6-integrin or FGFR1 were blocked individually with their respective specific siRNA, whereas the combination of both siRNA with a dual transfection led to a significantly higher inhibition of spheres formation (Figure 6D,E). In accordance with the inhibition of cell proliferation observed in Figure 4E, spheres size was also decreased with the dual transfection α6-integrin/FGFR1 (Figure 6F,G). In addition, the blocking of ZEB1 or YAP1 expression by specific siRNA similarly decreased neurospheres formation and spheres size (Figure S3B,C).

### 2.5. α6-Integrin Expression Correlates with the Target Genes ZEB1, YAP1, FGFR1, and FOXM1

The glioblastoma database of the Cancer Genome Atlas, (TCGA, *n* = 539) was used to analyze the correlations between α6-integrin expression and several potential target genes including ZEB1, YAP1, FGFR1, and FOXM1. As shown in Table 1, a significant positive correlation was observed between α6-integrin expression and each of the target genes tested.

Except for FOXM1, these results were confirmed in the Rembrandt database (*n* = 184) (Table 2).

### 2.6. High Expression of the Five Genes Signature: α6-Integrin/ZEB1/YAP1/FGFR1/FOXM1 Is Prognostic of the Overall Survival of GBM Patients

**Figure 7:**
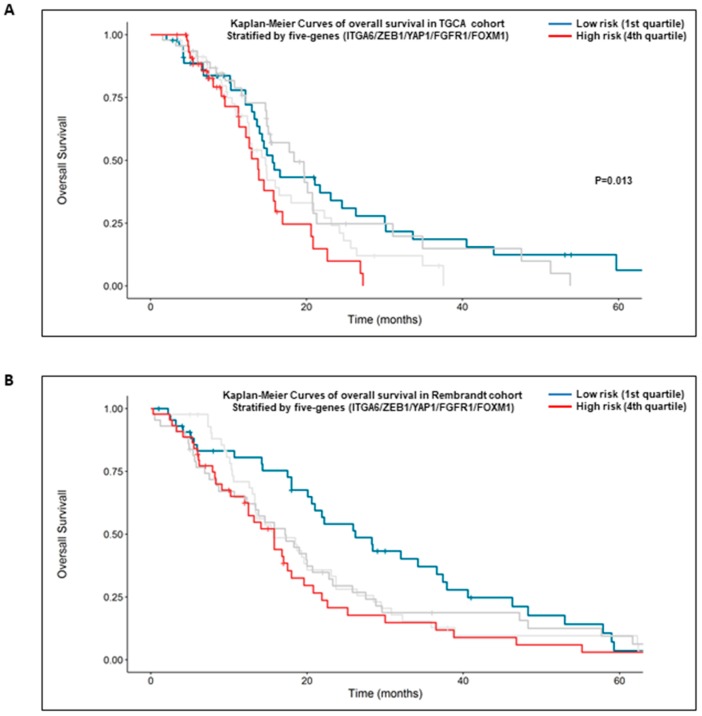
High expression of the five genes signature: α6-integrin/ZEB1/YAP1/FGFR1/FOXM1 is prognostic of survival of GBM patients. Kaplan-Meier curves depict the probability of overall survival based on the expression of a five genes signature: α6-integrin/ZEB1/YAP1/FGFR1/FOXM1. Analysis was performed from TGCA (**A**) or Rembrandt (**B**) databases. Patients were stratified by the upper or lower quartiles and the statistical analysis performed as described in section “Materials and Methods”.

Finally, we queried if the five genes signature, α6-integrin/ZEB1/YAP1/FGFR1/FOXM1, correlates with the survival of GBM patients. For this purpose, we analyzed two different databases, TCGA and Rembrandt. In the TGCA database, analyses were performed on 184 patients treated with standard radio-chemotherapy for primary GBM without prior glioma history (*n* = 184). Although the risk score was not found to be significantly associated with overall survival as a continuous variable (Hazard Ratio (HR) = 2.72 (0.97; 7.61), *p* = 0.057), the five genes signature was significantly associated with overall survival when patients samples were stratified by the highest and lowest quartile (high versus low risk: HR = 1.99 (1.16; 3.44), *p* = 0.013, Figure 7A).

In the Rembrandt database, the risk score and risk groups based on the same five genes were found to be significantly associated with the overall survival survival (HR = 2.49 (1.05; 5.89), *p* = 0.038; 4th quartile vs. 1st quartile: HR = 1.86 (1.16; 2.95), *p* = 0.009, Figure 7B), indicating that higher levels of this five-genes signature predicted significantly shorter overall survival as compared to patients with lower expression.

## 3. Discussion

GBM are known to be particularly aggressive and resistant to anti-cancer therapies, likely due to the presence of GBMSC. The FGFR/FGF axis is dysregulated in many cancers including GBM and represents an attractive target in clinical oncology because FGF signaling is involved in various aspects of cancer biology, including stemness, proliferation, invasion, angiogenesis, and drug resistance [11]. In particular, the axis FGFR1/FGF2 is crucial for GBMSC [18,19,20,21]. α6-integrin, which is overexpressed in GBMSC and contributes to maintaining their stem cell state, is also a potential therapeutic target to overcome GBM resistance to therapies. Considering that α6-integrin and FGFR1 are both involved in cancer stem cell biology of GBM, we analyzed the possibility that they coordinately regulate GBMSC. To our knowledge, a cross-talk between α6-integrin and FGFR1 has never been described. Our study adds an important mechanistic understanding of how α6-integrin and FGFR1 work in concert in GBMSC. In this study, we show for the first time that α6-integrin regulates the expression of FGFR1 and its target gene FOXM1 through an ERK-dependent mechanism. The ERK pathway is a common signaling pathway downstream of FGFR1 and α6-integrin that can be amplified by α6-integrin, which activates the ERKs directly or indirectly through the regulation of FGFR1 expression. Very few studies have reported a cross-talk between FGFRs and integrins. In endothelial cells, vitronectin increases FGFR1 and FGFR2 expression likely through the activation of αVβ3 and αVβ5 integrins [28]. In addition, two studies have shown that FGF1 and FGF2 can directly bind αvβ3 to induce endothelial cell proliferation and motility [29,30]. An intracellular cross-talk has also been reported in endothelial cells between integrins and FGFR1. Fibronectin, through the activation of β1 or β3, has been shown to trans-activate FGFR1 by phosphorylation via the non-receptor tyrosine kinase Src [31]. Finally, in breast cancer cells, α3-integrin has been reported to physically disrupt the interaction between FGFR1 and E-cadherin, increasing the metastatic process [32].

We recently identified the transcription factor ZEB1 as a downstream target of α6-integrin signaling, dependent on ERK activation [22]. Here we show that α6-integrin might control FGFR1 expression via ZEB1 since we observed a high inhibition of FGFR1 expression when ZEB1 was blocked by an siRNA. To our knowledge, the regulation of FGFR1 expression by ZEB1 has never been reported. However, few studies using Affymetrix-based expression have shown a positive correlation between ZEB1 and FGFR1 [23,33,34]. ZEB1 is known as a transcriptional repressor of epithelial genes; however, a recent paper by Lehmann [26] has reported that ZEB1 switches its function to a transcriptional co-activator by interacting with YAP1 in aggressive cancers. Our results suggest that α6-integrin regulates FGFR1 expression via the two partners of the ZEB1/YAP1 complex. As observed for ZEB1, we report not only a decrease of YAP1 expression when α6-integrin is blocked, but also a decrease in FGFR1 expression when YAP1 is inhibited. The regulation of YAP1 expression by α6-integrin has never been reported. However, in several cancers, integrins have been linked to YAP1 expression as well as YAP1 activation by nuclear translocation of the protein. In particular, α3-integrin has been shown to regulate YAP1 expression in GBM [35], whereas α1-integrin has been involved in YAP1 nuclear relocation in hepatocarcinoma [36]. Very few studies have reported the regulation of FGFR expression by YAP1. In lung cancer, YAP1 increases FGFR1 expression, and in cholangiocarcinoma, cross-talk between YAP1 and FGFR1, 2 and 4 has been shown [37,38]. 

Our results were in accordance with the positive correlation observed between α6-integrin expression and its target genes ZEB1, YAP1, FGFR1, and FOXM1 in GBM from two different databases, including respectively 539 and 184 patients. In addition, consistent with our pre-clinical data in vitro, the analyses from two distinct clinical datasets, TGCA and Rembrandt databases, demonstrate that GBM patients with high levels of the five genes signature, including α6-integrin and its targets, ZEB1, YAP1, FGFR1, and FOXM1, have a significantly shorter overall survival as compared to patients with lower expression.

Finally, when α6-integrin or FGFR1 were blocked individually with specific siRNA, we observed the same inhibition of FOXM1 target genes and stemness-related markers, as well as a similar decrease in GBMSC neurospheres forming capacity and spheroids growth. Interestingly, the combination of both siRNA with a dual transfection led to a significantly higher inhibition of spheres formation, suggesting that co-administration of anti-FGFR1 and anti-α6-integrin could provide an improved therapeutic response.

## 4. Materials and Methods

### 4.1. GBM Patient-Derived Cells

All GBM specimens were obtained after written informed consent from patients admitted to the Neurosurgery Department at Toulouse University Hospital under a clinical protocol (PI Pr. E. Cohen-Jonathan-Moyal) approved by the Human Research Ethics Committee (ethical code 12TETE01, ID-RCB number 2012-A00585-38, date of approval: 07-05-2012). Patient brain tumor samples were classified as GBM based on the World Health Organization (WHO). Primary neurospheres were maintained in DMEM-F12 (GIBCO, Life Technologies, Courtaboeuf, France) supplemented with B27 and N2 (Life Technologies, Courtaboeuf, France), 25 ng/mL of FGF-2 and EGF (Peprotech, Neuilly sur Seine, France) at 37 °C in a 5% CO_2_ humidified incubator and cultured during less than 12 passages to avoid loss of cell characteristics.

### 4.2. SiRNA Transfection, RNA Extraction, Reverse Transcription, and Real-Time PCR

The siRNA directed against α6-integrin, FGFR1, ZEB1, YAP1 or the scramble control were purchased from Qiagen (Courtaboeuf, France) and transfected using Lipofectamine RNAi Max (Invitrogen, Courtaboeuf, France) following the manufacturer protocol. Total RNA was isolated by the RNeasy RNA isolation Kit (Qiagen) then reverse transcribed using the RT transcription kit Prime Script RT Reagent kit (TAKARA, Ozyme, Saint Quentin en Yvelines, France). mRNA expression was determined with real-time PCR, using the ABI-Stepone+ (Applied Biosystems, Villebond sur Yvette, France). GAPDH was used for normalization. Experiments were performed independently 3 times in triplicates on GBMSC neurospheres at 3 different passages between passages 4 and 12. 

### 4.3. D Spheroid Formation

Cells derived from GBM biopsy specimens, transfected with a specific α6-integrin siRNA, a specific FGFR1 siRNA or both were seeded in 96 wells plates (100 cells/well). After 8–10 days, the number of neurospheres/wells was counted under the microscope. The spheres area was measured using the Image J software (version 1.49V, free download at www.01net.com) by using the scale bar obtained with the Nikon software NIS Elements (version 4.0, Nikon, Champigny sur Marne, France). Experiments were performed independently 3 times with 12 wells/conditions on GBMSC neurospheres at 3 different passages between passages 4 and 12. 

### 4.4. Western-Blot Analysis

Identical levels of proteins were separated by SDS-PAGE and analyzed by Western-blot with the indicated antibodies. Primary antibodies used for western blot: Tuj-1, OLIG2 (Abcam, Cambridge, United Kingdom), Actin, (Millipore, Molsheim, France), FGFR1, FOXM1, ZEB1, FRS2, YAP1, 12hosphor-FRS2, 12hosphor-FAK, 12hosphor-ERKs, 12hosphor-AKT, 12hosphor-JNK (Cell Signaling). Secondary antibodies used: Immunopure goat anti-rabbit IgG (H + L) peroxidase-conjugated and rabbit anti-mouse IgG (H + L) peroxidase-conjugated (Thermo Scientific, Life Technologies, Courtaboeuf, France). Western blots were revealed using “ECL Revelblot Substrate Chemoluminescent” (Ozyme, Saint Quentin en Yvelines, France). Western blot experiments were performed independently 3 times on GBMSC neurospheres at 3 different passages between passages 4 and 12. 

### 4.5. Cell Cycle Analysis

24 h post-transfection, cells were fixed in 70% ice-cold ethanol for 1H at 4 °C. After washing, the cell pellet was resuspended in propidium iodide (PI)-staining buffer (50 μg/mL PI, 10 μg/mL RNAse A) and incubated for 15 min at 37 °C. The DNA content was analyzed by flow cytometry (BD AccuriTM C6 cytometer). The % of cells in sub-G1, G1, S and G2/M phases were quantified with the BD Accuri C6 software (Le Pont de Claix, France). Experiments were performed independently 3 times in duplicates on GBMSC neurospheres at 3 different passages between passages 4 and 12. 

### 4.6. Statistical Analysis for In Vitro Studies

For cell cycle analysis, spheres number, and spheres size, control samples and siRNA samples were compared using the unpaired Student’s *t*-test using Excell software. For mRNA, Western blot quantifications, and cell number counting, the control condition was set to 1, and Student’s *t*-test with Welch’s correction for unequal variances was performed using Excell software.

### 4.7. Genes Correlations in TGCA and Rembrandt Databases

The glioblastoma database of the Cancer Genome Atlas, (TCGA, *n* = 539) (https://genome-cancer.ucsc.edu/) and Rembrandt database (*n* = 184) (http://www.betastasis.com/glioma/rembrandt/) were used to analyze the correlations between α6-integrin expression and several potential target genes including ZEB1, YAP1, FGFR1, and FOXM1. Links between biomarkers were assessed using Spearman’s rank correlation coefficient and tests were considered significant at alpha 5% level. Statistical analyses were performed using R. 3.5.1 software (free download at www.01net.com).

### 4.8. Association Between Five Genes: Integrin α6/ZEB1/YAP1/FGFR1/FOXM1 and Overall Survival in GBM Patients

Survival analyses were performed on the glioblastoma database of The Cancer Genome Atlas (https://genome-cancer.ucsc.edu/), on patients treated with standard radio-chemotherapy for primary GBM, excluding patients with prior glioma history (*n* = 184). Overall survival was estimated using the Kaplan-Meier method. A risk score was created based on the linear predictor given by the multivariable Cox proportional hazard model on overall survival using a set of 5 genes (α6-integrin/ZEB1/YAP1/FGFR1/FOXM1). This score was then divided into four groups by taking the quartiles. To confirm the prognostic ability of our risk score, coefficients found on the TCGA database were applied on REMBRANDT patients (http://www.betastasis.com/glioma/rembrandt/).

## 5. Conclusions

Our results led us to a novel signaling model in GBMSC where α6-integrin, through the transcription factors ZEB1 and YAP1, increases FGFR1 transcription and its downstream targets, resulting in enhanced expression of stemness-related factors as well as an increase in spheres forming capacity and spheroids growth. In addition, the five-gene signature including α6-integrin, ZEB1/YAP1, FGFR1, and its downstream target FOXM1 is prognostic of the overall survival of patients with GBM. These data suggest that co-administration of anti-FGFR1 and anti-α6-integrin could provide an improved therapeutic response.

## Figures and Tables

**Table 1:** Inter-genes correlations in the TGCA database (*n* = 539). Values correspond to Spearman’s rank correlation coefficients and their associated *p*-values *** *p* < 0.001; ** 0.001 < *p* < 0.01; * 0.01 < *p* < 0.05.

Genes	ITGA6	FOXM1	FGFR1	ZEB1	YAP1
**ITGA6**	1.0000				
**FOXM1**	0.1215 ** *p* = 0.0047	1.0000			
**FGFR1**	0.2610 *** *p* < 0.0001	0.2543 *** *p* < 0.0001	1.0000		
**ZEB1**	0.2611 *p* < 0.0001	0.2810 *** *p* < 0.0001	0.3414 *** *p* < 0.0001	1.0000	
**YAP1**	0.4111 *** *p* < 0.0001	0.0548 *p* = 2042	0.3145 *** *p* <0.0001	0.1579 ** *p* = 0.0002	1.0000

**Table 2:** Inter-genes correlations in the Rembrandt database (*n* = 184). Values correspond to Spearman’s rank correlation coefficients and their associated *p*-values *** *p* < 0.001; ** 0.001 < *p* < 0.01; * 0.01 < *p* < 0.05.

Genes	ITGA6	FOXM1	FGFR1	ZEB1	YAP1
**ITGA6**	1.0000				
**FOXM1**	0.1307 *p* = 0.077	1.0000			
**FGFR1**	0.1662 * *p* = 0.0241	0.3943 *** *p* < 0.0001	1.0000		
**ZEB1**	0.2141 ** *p* = 0.0035	0.4381 *** *p* < 0.0001	0.2492 *** *p* = 0.0006	1.0000	
**YAP1**	0.5133 *** *p* < 0.0001	0.2296 ** *p* = 0.0017	0.3958 *** *p* < 0.0001	0.1960 ** *p* = 0.0077	1.0000

## Supplementary Materials

The following are available online at https://www.mdpi.com/2072-6694/11/3/406/s1, Figure S1: GBMSC derived from GBM biopsy specimens (GC1, GC2) were transfected with an α6-integrin siRNA (si-α6 (1) or si-α6 (2)), a FGFR1 siRNA (si-FGFR1), a combination of both siRNA (si-α6 (1) + si-FGFR1), a scramble control (si-Scr) or a ZEB1 siRNA (si-ZEB1 (2)) as indicated, Figure S2: (A) GBMSC derived from GBM biopsy specimens (GC1, GC2) were transfected with a ZEB1 siRNA (si-ZEB1 (1)) or a scramble control (si-Scr), Figure S3: GBMSC derived from GBM biopsy specimens (GC1, GC2) were transfected with a ZEB1 siRNA (si-ZEB1(1)), a YAP1 siRNA (si-YAP1(1)) or a scramble control (si-Scr).
