# Supplementary material for: Alpha6-Integrin Regulates FGFR1 Expression through the ZEB1/YAP1 Transcription Complex in Glioblastoma Stem Cells Resulting in Enhanced Proliferation and Stemness
Source: Cancers (Basel). 2019 Mar 22;11(3):406. doi: 10.3390/cancers11030406 (PMC6468800; doi:10.3390/cancers11030406)
Supplement: Supplementary file 1 [file cancers-11-00406-s001.pdf]

cancers

MDPI

Supplementary Materials: Alpha6-Integrin Regulates FGFR1 Expression Through the ZEB1/YAP1 Transcription Complex in Glioblastoma Stem Cells Resulting in Enhanced Proliferation and Stemness

Aline KOWALSKI-CHAUVEL, Valerie GOUAZE-ANDERSSON, Laurent BARICAULT, Elodie MARTIN, Caroline DELMAS, Christine TOULAS, Elizabeth COHEN-JONATHAN-MOYAL and Catherine SEVA

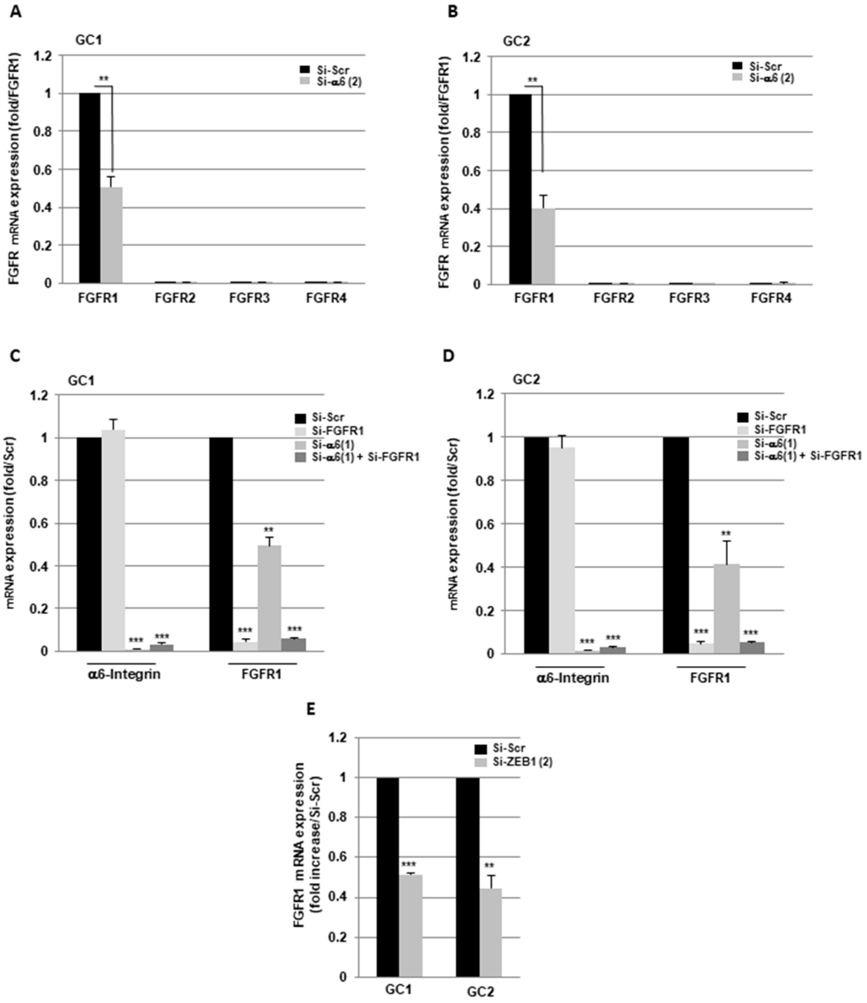

Figure S1. (**A**–**E**) GBMSC derived from GBM biopsy specimens (GC1, GC2) were transfected with an α6-integrin siRNA (si-α6 (1) or si-α6 (2)), a FGFR1 siRNA (si-FGFR1), a combination of both siRNA (si–α6 (1) + si-FGFR1), a scramble control (si-Scr) or a ZEB1 siRNA (si-ZEB1 (2)) as indicated. FGFRs or α6-integrin expression was analyzed by real time PCR. Quantifications of 3 independent experiments are presented as means ± SD. *** $p < 0.001$; ** $0.001 < p < 0.01$.

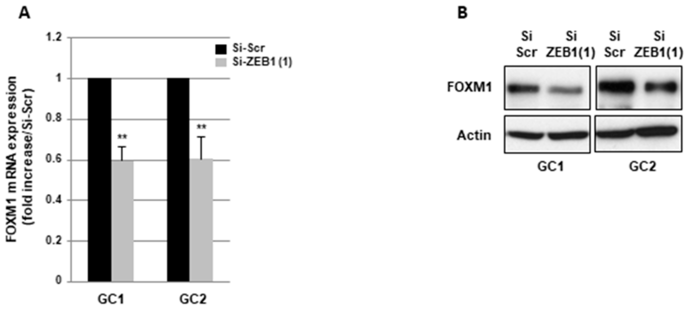

Figure S2. (**A**,**B**) GBMSC derived from GBM biopsy specimens (GC1, GC2) were transfected with a ZEB1 siRNA (si-ZEB1 (1)) or a scramble control (si-Scr). (**A**) FOXM1 expression was analyzed by real time PCR. Quantifications of 3 independent experiments are presented as means ± SD. ** 0.001 < p < 0.01. (**B**) FOXM1 protein expression was analyzed by western blot. Images are representative of 3 independent experiments. Actin was used as a loading control.

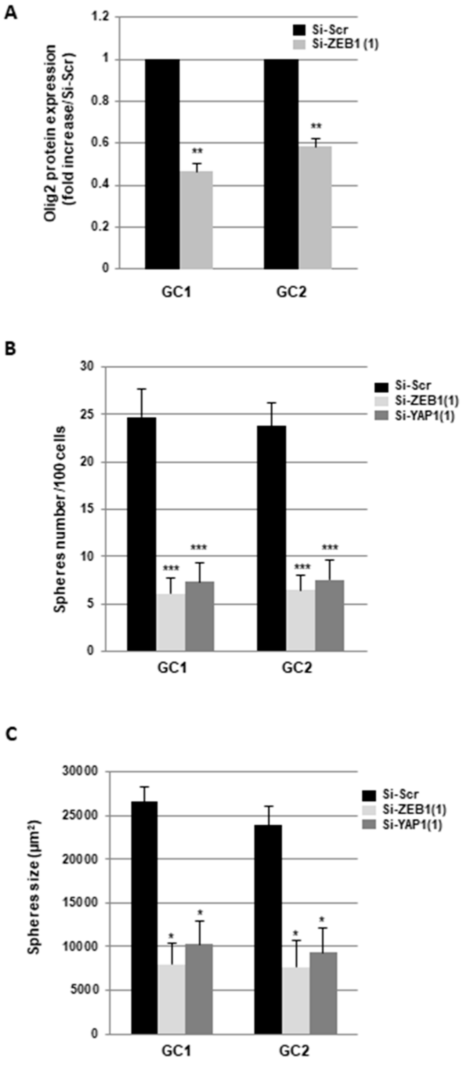

Figure S3. GBMSC derived from GBM biopsy specimens (GC1, GC2) were transfected with a ZEB1 siRNA (si-ZEB1(1)), a YAP1 siRNA (si-YAP1(1)) or a scramble control (si-Scr). (**A**) Olig2 protein expression was analyzed by western blot. (**B**,**C**) Spheres formation was analyzed as described in "methods". (**B**). Neurospheres number was counted under the microscope. (**A**,**C**) Western blot images and the spheres size were quantified using the Image J software. Quantifications of 3 experiments are presented as means ± SD. *** p < 0.001; ** 0.001 < p < 0.01; * 0.01 < p < 0.05.

© 2019 by the authors. Licensee MDPI, Basel, Switzerland. This article is an open access article distributed under the terms and conditions of the Creative Commons Attribution (CC BY) license (http://creativecommons.org/licenses/by/4.0/).
